# A phantom study of internal target volume and mid-position accuracy in adaptive and conventional four-dimensional computed tomography across regular and irregular motion

**DOI:** 10.1016/j.phro.2025.100845

**Published:** 2025-10-01

**Authors:** Bart J.J. Kremers, Dave S.C. van Gruijthuijsen, Dominique Reijtenbagh, Jacco L.G. Steenhuijsen, Mariska de Smet, Rob H.N. Tijssen

**Affiliations:** a Department of Radiation Oncology, Catharina Hospital Eindhoven, Eindhoven, Netherlands (the); b Maastro Clinic, Maastricht, Netherlands (the); c Department of Biomedical Engineering, Technical University Eindhoven, Eindhoven, Netherlands (the)

**Keywords:** 4DCT quality assurance, Adaptive 4DCT, Mid-position

## Abstract

This technical note evaluates the performance of an adaptive four-dimensional computed tomography (4DCT) acquisition method compared to conventional 4DCT using a motion phantom. Metrics assessed include deviations in volume, CT number, diameter, peak-to-peak amplitude and determination of the internal target volume (ITV) and mid-position. Under regular breathing, most measurements fall within predefined clinical tolerances for all systems. Under irregular motion, the adaptive method showed reduced deviations in ITV and minimal impact on mid-position determination. These findings support the clinical value of adaptive 4DCT in improving motion management and target definition accuracy in radiotherapy planning.

## Highlights

• Adaptive 4DCT reduces ITV deviations under irregular breathing conditions.
• Mid-position accuracy remains robust across adaptive and conventional acquisition techniques.
• Adaptive 4DCT shows superior motion fidelity compared to conventional 4DCT.
• Conventional 4DCT exhibits greater variability in volume and amplitude metrics.

## Graphical abstract

**Figure:**
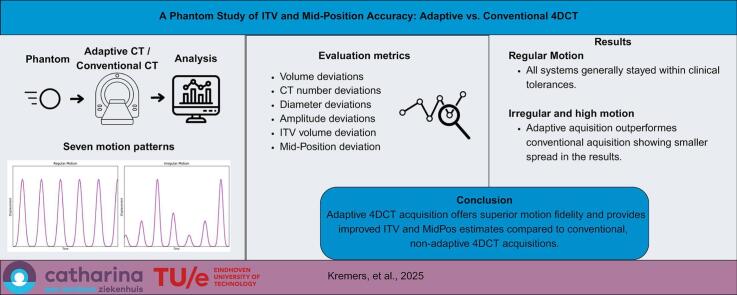

## 1 Introduction

In modern radiotherapy, respiratory-induced tumor and organ motion introduces positional uncertainties that can compromise treatment accuracy. Four-dimensional computed tomography (4DCT) mitigates these uncertainties by capturing motion throughout the respiratory cycle [[1], [2], [3], [4]].

Accurate tumor localization is essential for determining the planning target volume (PTV). Wolthaus et al. [[5], [6], [7]] proposed two 4DCT-based strategies, mid-position (MidPos) and mid-ventilation (MidVent), which allow for smaller PTV margins compared to the traditional internal target volume (ITV) approach. While ITV encompasses the full tumor excursion, MidPos is the mathematical time-weighted mean tumor position. The MidVent position corresponds to the geometrically nearest acquired 4DCT phase. Due to tumor motion hysteresis, the MidPos and MidVent positions may differ.

Conventional 4DCT is prone to motion artifacts, particularly when patients exhibit irregular breathing [[8], [9], [10]]. To address this, Werner et al. [11] proposed an adaptive acquisition method, that adjusts to breathing patterns and optimises binning points, thereby reducing artifacts [10].

Quality assurance in 4DCT imaging for radiotherapy is essential as emphasized in multicenter trials such as the ROSEL study [12]. Several studies have reported on quality assurance of adaptive 4DCT performance [[13], [14], [15]]. However, the impact of adaptive motion management on ITV and MidPos (or MidVent) determination has not been thoroughly investigated.

This study compares an adaptive 4DCT algorithm with conventional 4DCT algorithms and evaluates their performance and impact on ITV and MidPos determination, to identify advantages and limitations of adaptive 4DCT in clinical practice.

## 2 Materials and methods

### 2.1 4DCT imaging systems, phantom and breathing curves

Two 4DCT imaging systems were evaluated;

1) Philips Brilliance Big Bore (Philips Medical Systems, Best, the Netherlands), using a conventional spiral acquisition mode. This system, hereafter referred to as Philips conventional (PConv), operated with a pitch of 0.081 for periods <6 s and 0.06 for periods ≥6 s, in accordance with our clinical protocol to prevent undersampling.
2) Siemens SOMATOM go.Open Pro (Siemens Healthineers AG, Forchheim, Germany), evaluated using two scanning algorithms:
  • Direct i4D, an adaptive sequential acquisition algorithm, referred to as Siemens Direct i4D (Si4D).
  • Conventional spiral acquisition mode, with a pitch of 0.07, referred to as Siemens conventional (SConv).

Both systems used a tube voltage of 120 kV, and CT reconstructions were made with a 2 mm slice thickness.

The Philips scanner used the vendor-provided pneumatic bellows system, which detects respiratory motion through pressure changes in a belt wrapped around the patient’s abdomen [16]. On the Siemens scanner, the Anzai belt (Anzai Medical, Tokyo, Japan) was used, which similarly measures abdominal pressure variations but with a different sensor configuration and signal processing approach [17]. Both systems serve as external surrogates for respiratory motion, enabling sorting of 4DCT data. For each acquisition, ten respiratory phases were reconstructed using phase sorting, which is required to calculate the time-weighted mean position [6]. Scan length was manually selected from a scout image to ensure full coverage of the spherical target’s motion range.

The QUASAR™ MRI^4D^ Motion Phantom was used for all experiments. A 30 mm spherical target located in the hollow acrylic insert, was filled with a 1:2 dilution of Telebrix Gastro (300 mg Iodine/ml and demineralized water. This resulted in average CT numbers of 2038 HU on the Philips system and 1997 HU on the Siemens system, providing sufficient contrast against the acrylic stem for full width at half maximum analysis. The tube housing was left unfilled, and the insert was centrally positioned. A firm block of Styrofoam was placed against the top of the tube and clamped to the phantom using the respiratory belts, which were wrapped around the phantom to monitor tube motion. Fig. S1 shows the setup on the Siemens CT with the Anzai belt.

One-dimensional breathing patterns in the superior-inferior (SI) direction based on a cos^6^ function were selected from previous studies by Tahiri et al. and Szkitsak et al. [13,14], which included four regular and three irregular breathing patterns. Details on amplitude and period can be found in the supplementary material Fig. S2 and Table S1. All irregular breathing patterns, began with a one-minute regular signal to allow the Si4D system to analyze and optimize scan settings (FAST 4D) [10]. 4DCT acquisition on all modalities was always started after this period. Each breathing pattern was measured three times for each acquisition mode, resulting in a total of 63 measurements.

Additionally, one acquisition per system was performed without phantom movement, using a clinical 3D abdomen protocol. These are referred to as the static reference condition.

### 2.2 Image processing, auto-contouring and evaluation metrics

All 4DCT and static images were imported into RayStation 12A (RaySearch Laboratories AB, Stockholm, Sweden) for processing. All 4DCT phase images were auto-contoured using a Hounsfield Unit (HU) threshold-based method [15]. Thresholds were determined based on the static reference images.

From each contour, the following parameters were extracted: 1) target volume, 2) average CT number (contour cropped 2 mm inward to reduce edge effects), 3) diameter in the SI direction, and 4) the extreme phases in order to determine the peak-to-peak amplitude of the center of mass. The ITV contour was generated by performing the union of the 10 individual respiratory phase contours.

Definitions of the deviation in volume, average CT number, diameter in SI direction, peak-to-peak amplitude and the ITV following Tahiri et al. [13], along with their corresponding tolerances, based on Canadian Partnership for Quality Radiotherapy (CPQR) guidelines [16] for regular motion and Tahiri et al. for irregular motion [13], are provided in the supplementary material Table S2.

For irregular motion patterns, deviations in amplitude and ITV were calculated using the amplitude from the initial (regular) learning phase as the reference, i.e., the amplitude during the first minute of the signal.

To quantify the impact of different 4D acquisition techniques on our clinically used MidPos determination, we used our in-house developed script implemented in RayStation. The script starts with selecting a region-of-interest (ROI) contour, for which in this analysis the previously created threshold contour on the 0 % phase was used. Then a registration focus ROI was generated by expanding this contour by +2 cm in all directions. Next, a deformable registration was performed from the 0 % phase to all other phase scans focusing on the area within this focus ROI. The script then maps the original ROI onto each phase scan based on this registration. These mapped contours were subsequently used to determine the MidPos by averaging the center of mass position of these contours. Mapped contours were chosen over threshold-based contours to better reflect clinical practice. A visual inspection was conducted to compare the threshold-based and deformably registered contours, and no noticeable differences were observed. The evaluation metrics MidPos deviation is given in the supplementary material Table S2.

## 3 Results

**Fig. 1:**
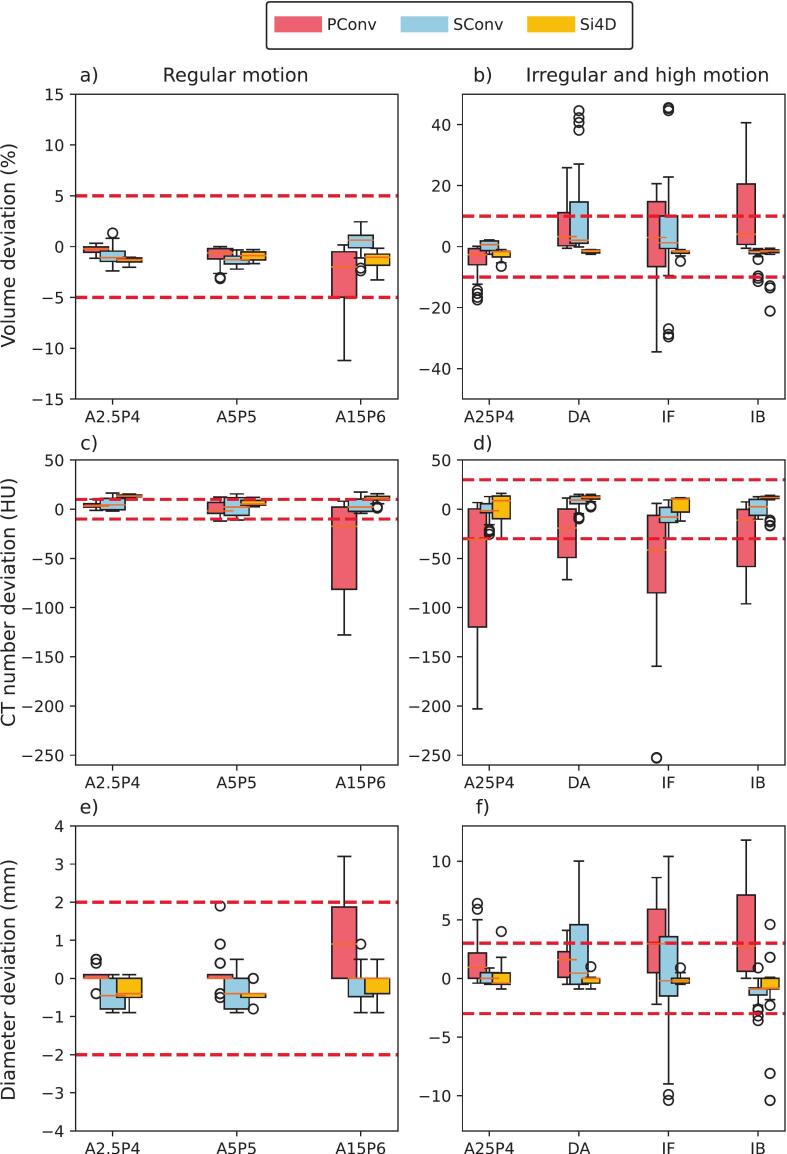
Box-plots showing data from three individual measurements across ten respiratory phases (30 data points per signal and modality). Data are presented for the PConv (red), SConv (blue), and Si4D (yellow) systems. Red dashed lines indicate predefined tolerance thresholds. a & b) volume deviation, c & d) average CT number deviation and e & f) diameter deviation. Note that the y-axis scales differ for the regular curves (left column) and irregular/highly-moving curves (right column). The x-axis denotes the applied breathing curves. (For interpretation of the references to colour in this figure legend, the reader is referred to the web version of this article.)

For regular motion, SConv and Si4D exhibit minimal volume deviations, staying within the tolerance range. PConv shows a notable underestimation in the condition with a peak-to-peak amplitude of 15 mm (signal A15P6) (Fig. 1a). For irregular/high-motion conditions, where high-motion is defined as a signal with the combination of a four seconds breathing cycle with 25 mm amplitude [13], volume deviations become more pronounced across all modalities, with PConv and SConv demonstrating the largest spread, particularly for the double amplitude (DA) and irregular frequency (IF) waveforms. Si4D remains within the ±10 % tolerance range, except for a few outliers in the irregular breathing (IB) signal (Fig. 1b).

Under regular motion SConv and Si4D demonstrate stable performance in the CT number deviation, remaining mostly within tolerance. PConv, on the other hand, shows a considerable underestimation when the peak-to-peak amplitude is increased to 15 mm (signal A15P6) (Fig. 1c). With irregular/high-motion all modalities exhibit increased variability in the CT number deviation, PConv demonstrating the most significant deviations, frequently falling outside tolerance limits. While SConv and Si4D remain more stable, some deviations still exceed the defined tolerance range (Fig. 1d).

Under regular motion, all three modalities generally remain within diameter deviation tolerance, except for the measurements where the peak-to-peak amplitude is increased to 15 mm (signal A15P6) for PConv (Fig. 1e). Under irregular/high-motion, PConv and SConv exhibit a broader variability in diameter deviation, often exceeding the set tolerance. Si4D, however, remains largely within tolerance, except for a few outliers in the IB signal (Fig. 1f).

**Fig. 2:**
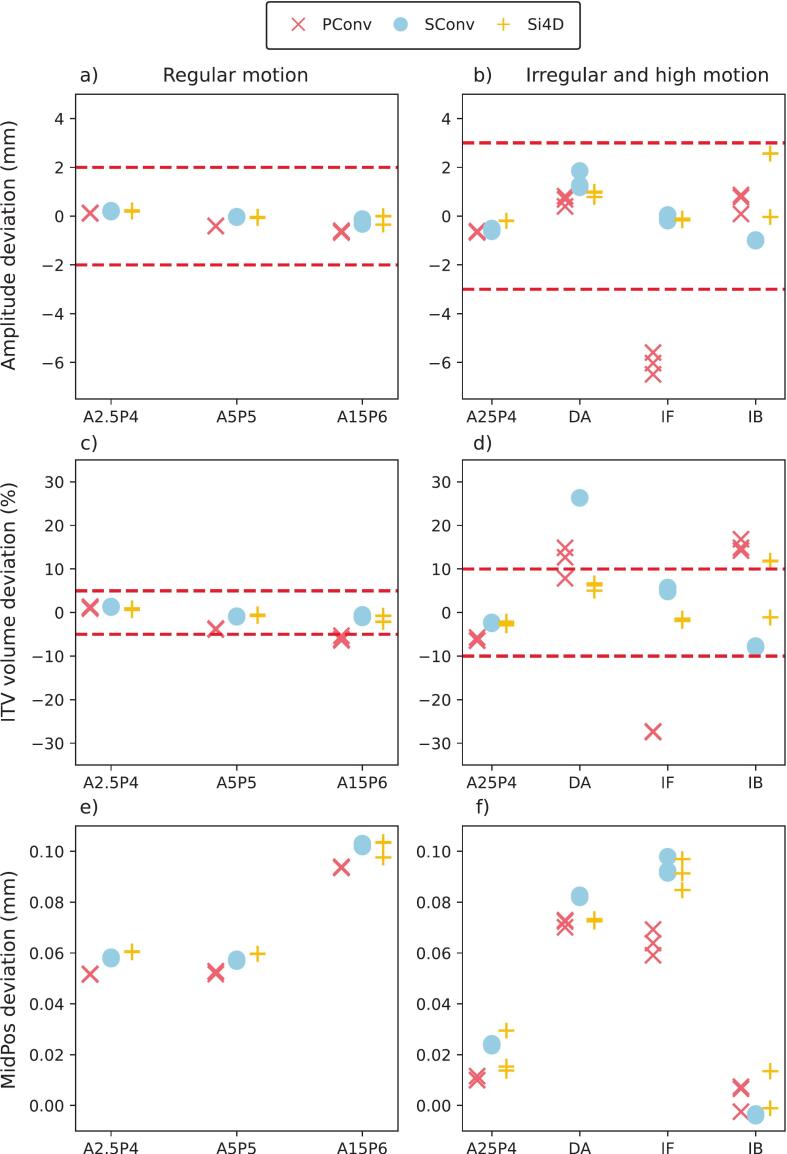
Deviations in amplitude (a, b), ITV volume (c, d) and MidPos (e, f) in regular and irregular/high-motion conditions. The red ‘x’, blue 'o' and yellow '+' represent individual measurements on the PConv, SConv and Si4D systems respectively. The red dashed lines indicate tolerance thresholds, note that these differ for regular and irregular/ high-motion. The x-axis denotes the applied breathing curves. Note that in e and f the range of the y-axis is only 0.1 mm. (For interpretation of the references to colour in this figure legend, the reader is referred to the web version of this article.)

Under regular motion, amplitude deviations for all three modalities remain within tolerance of ±2 mm, with only minor deviations observed (Fig. 2a). For irregular/high-motion conditions, amplitude deviations become more pronounced, particularly for PConv, which exhibits substantial deviations in the IF case (Fig. 2b).

All points fall within the set ITV volume deviation tolerance of ±5 % except for the PConv modality for the motion with the peak-to-peak amplitude of 15 mm (A15P6) (Fig. 2c). For irregular/high-motion, PConv and SConv show ITV volume deviations up to −28 % and +27 % respectively, with SConv demonstrating a greater spread in results, particularly for the DA and IF signals. In contrast, Si4D consistently maintains shows smaller ITV deviations, except for IB (Fig. 2d).

The MidPos deviation remains below 0.15 mm across all acquisition techniques and motion scenarios, with a maximum deviation of 0.10 mm observed for Si4D under the A15P6 signal. Notably, the spread in measurements per signal and modality remains extremely small, with a maximum variation of only 0.01 mm for the IF signal in the PConv and Si4D system (Fig. 2e and Fig. 2f).

## 4 Discussion

This study assessed how different 4DCT acquisition algorithms affect ITV and MidPos determination. A detailed analysis on the image quality, CT number, target volume, amplitude, and diameter deviations was conducted with emphasis on performance under irregular respiratory motion.

All algorithms perform well under regular motion, with SConv and Si4D showing the most consistent results. Under irregular/high-motion conditions, variability increases, with PConv exhibiting the largest deviations. Si4D proved most robust, maintaining accuracy across nearly all scenarios. These results support the advantage of adaptive 4DCT (Direct i4D) in managing motion, particularly irregular breathing, enabling more precise target delineation and PTV margin definition consistent with Burghelea et al. [15]. In future work it might be useful to also include conformity indices into the evaluation [18]. The amplitude deviations (Fig. 2) further corroborate these results. Siemens systems remained within the tolerance range for most cases, while the Philips system exhibited substantial negative amplitude deviations under IB conditions. The i4D system, by adapting to breathing patterns, mitigated these issues. A notable spread in Si4D measurements under IB was linked to scan timing, one scan occurred during a low-amplitude phase, while others captured high-amplitude phases, leading to artifacts. This issue is due to the periodicity of the pattern and is not to be expected in clinical breathing patterns. The PConv and SConv systems, which employ spiral acquisition method, effectively mitigate these errors by averaging out variations over time and lead to a more smeared out image.

Si4D also showed the lowest ITV volume deviations, with the largest again under IB, likely due to the same timing issue. MidPos deviations remained minimal across all systems, even under irregular motion. The maximum deviation (0.10 mm for Si4D in A15P6) was well within clinical tolerance, confirming the robustness of adaptive 4DCT. Measurement spread was minimal, with a maximum variation of 0.01 mm (IF signal in PConv and Si4D), indicating high stability of MidPos determination across modalities. In conclusion, our findings indicate the adaptive 4DCT acquisition via Direct i4D offers superior motion fidelity and provides improved ITV and MidPos estimates compared to conventional, non-adaptive 4DCT acquisitions.

## CRediT authorship contribution statement

**Bart J.J. Kremers:** Conceptualization, Formal analysis, Methodology, Investigation, Visualization, Writing – original draft. **Dave S.C. van Gruijthuijsen:** Software. **Dominique Reijtenbagh:** Conceptualization, Investigation. **Jacco L.G. Steenhuijsen:** Conceptualization. **Mariska de Smet:** Conceptualization. **Rob H.N. Tijssen:** Conceptualization, Methodology, Supervision, Writing – review & editing.

## Declaration of competing interest

The authors declare that they have no known competing financial interests or personal relationships that could have appeared to influence the work reported in this paper.
