# Supplementary material for: A phantom study of internal target volume and mid-position accuracy in adaptive and conventional four-dimensional computed tomography across regular and irregular motion
Source: Phys Imaging Radiat Oncol. 2025 Oct 1;36:100845. doi: 10.1016/j.phro.2025.100845 (PMC12547255; doi:10.1016/j.phro.2025.100845)
Supplement: Supplementary Data 1 [file mmc1.docx]

Supplementary material: A Phantom Study of Internal Target Volume and Mid-Position Accuracy in Adaptive and Conventional Four-Dimensional Computed Tomography Across Regular and Irregular Motion

Bart J.J. Kremers^1^, Dave S.C. van Gruijthuijsen^1^, Dominique Reijtenbagh^1,2^, Jacco Steenhuijsen^1^, Mariska de Smet^1^, Rob H.N. Tijssen^1,3,*^

^1^ Department of radiation oncology, Catharina Hospital Eindhoven, Eindhoven, the Netherlands

^2^Maastro Clinic, Maastricht, The Netherlands

^3^Department of Biomedical Engineering, Technical University Eindhoven, Eindhoven, the Netherlands

*robtijssen@catharinaziekenhuis.nl

# Setup overview and typical examples


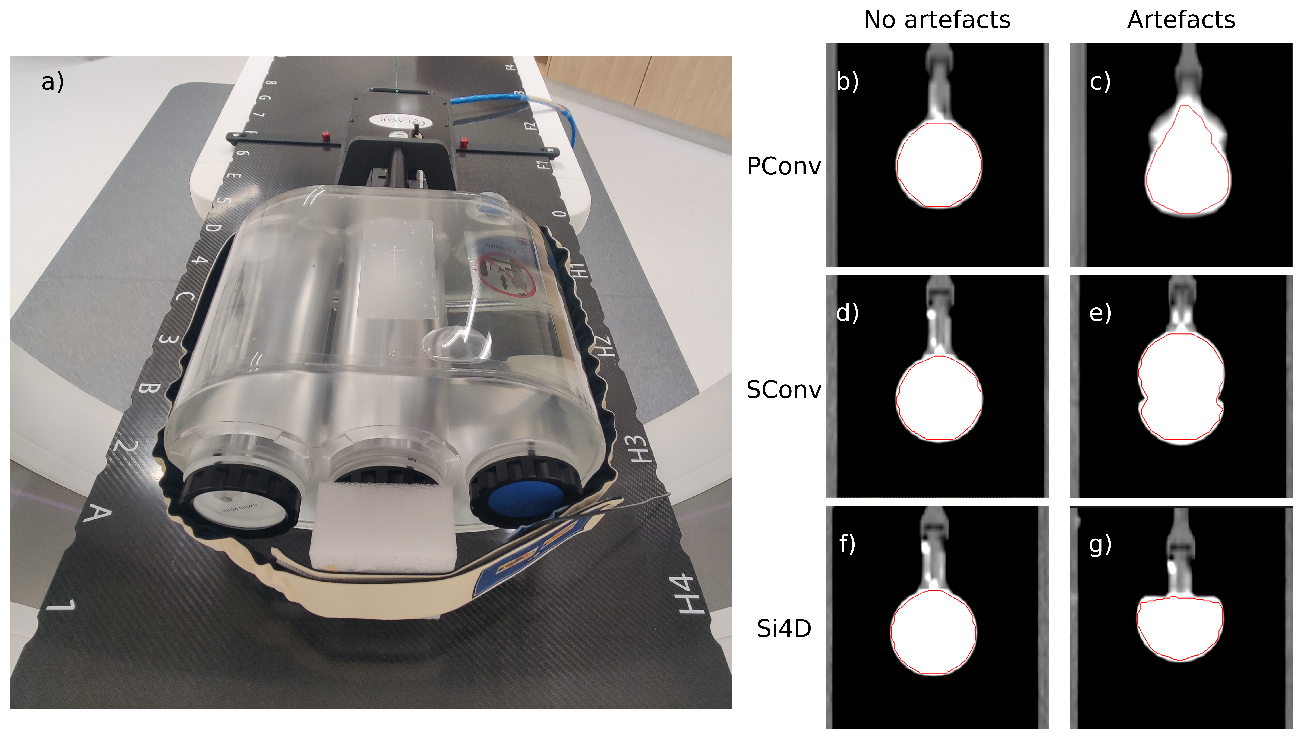


Figure S1: a) Overview of the setup of the QUASAR™ MRI⁴ᴰ Motion Phantom with a firm block of Styrofoam clamped to the phantom using the Anzai belt on the Siemens CT. b-g) Typical examples of the spherical target with and without artifacts for different modalities, Philips conventional (PConv), Siemens conventional (SConv) and Siemens Direct i4D (Si4D). The red line depicts the auto-contoured contours using the HU threshold method.

# Breathing curves


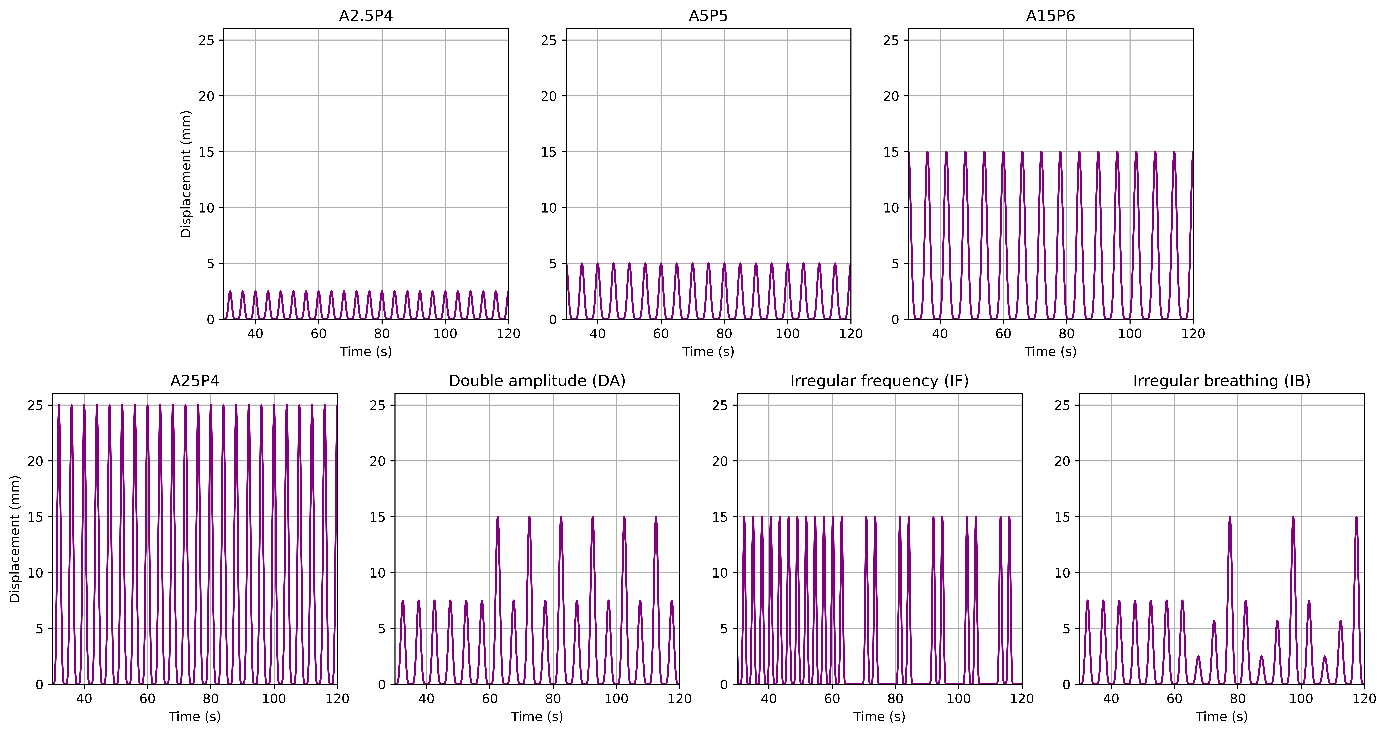


Figure S2: Breathing curves based on cos^6^ function supplied to QUASAR™ MRI^4D^ Motion Phantom, ranging from 2.5 mm up till 25 mm peak-to-peak amplitude. The first minute of the irregular curves has been made regular, this has been done to allow the Siemens CT system to analyze the breathing pattern and define a reference amplitude for adaptive acquisition and optimized binning. While the same signal is used in the conventional spiral 4DCT mode, actual image acquisition only begins after the learning period has passed.

Table S1: Overview of breathing curve parameters.

| **Motion type** | **Motion name** | **Abbreviation** | **Peak-to-peak amplitude (mm)** | **Period (s)** | **Time until signal switches (s)*** | **Peak-to-peak amplitude until signal switches (mm)** |
| --- | --- | --- | --- | --- | --- | --- |
| Regular motion | cos6A2.5P4 | A2.5P4 | 2.5 | 4 | - | - |
|  | cos6A5P5 | A5P5 | 5 | 5 | - | - |
|  | cos6A15P6 | A15P6 | 15 | 6 | - | - |
| Irregular and high motion | cos6A25P4 | A25P4 | 25 | 4 | - | - |
|  | Double amplitude | DA | 7.5/15 | 5 | 60 | 7.5 |
|  | Irregular frequency | IF | 15 | 2.8 pause of 5 | 60 | 15 |
|  | Irregular breathing | IB | 2.5/5.7/7.5/15 | 5.2 | 60 | 7.5 |

*Signal switch in order to go through initial learning phase of the Siemens CT system.

# Evaluation metrics and tolerances

Table S2: Overview of evaluation metrics and their tolerances.

| **Metric** | **Description** | **Tolerances** | | |
| --- | --- | --- | --- | --- |
| Volume deviation ΔV4DCT | $\Delta V_{4DCT}=100\%\frac{V_{4DCT}-V_{3DCT}}{V_{3DCT}}$ | < 5 % | 5 % < ΔV4DCT < 10 % | > 10 % |
| CT number deviation ΔHU4DCT | Mean CT number in contoured target, decreased with a margin of 2 mm to avoid CT number  fluctuations at the edge. | < 10 HU | 10 HU < ΔHU4DCT < 30 HU | > 30 HU |
| Diameter deviation ΔD4DCT | ΔD_4DCT_ = D_FWHM_ - D_ground-truth_ | < 2 mm | 2 mm < ΔD4DCT < 3 mm | > 3 mm |
| Amplitude deviation ΔA4DCT | ΔA_4DCT_ = A_4DCT_ - A_ground-truth_ | < 2 mm | 2 mm < ΔA4DCT < 3 mm | > 3 mm |
| ITV volume deviation ΔVITV | $\Delta V_{ITV}=100\%\cdot\left( \frac{V_{ITV,Raystation}-V_{ITV,GroundTruth}}{V_{ITV,GroundTruth}} \right)$ where $V_{ITV,GroundTruth}=A_{signal}\pi r^{2}+\frac{4}{3}\pi r^{3}$ | < 5 % | 5 % < ΔVITV < 10 % | > 10 % |
| MidPos deviation ∆xMidPos | $\Delta x_{MidPos}=x_{MidPos,script}-x_{MidPos,signal}$ | - | - | - |

# Examples of target per phase scan

Table S3: individual phases for A5P5 breathing curve acquired on Pconv, Sconv and Si4D.

| A5P5 | 0% | 10% | 20% | 30% | 40% | 50% | 60% | 70% | 80% | 90% |
| --- | --- | --- | --- | --- | --- | --- | --- | --- | --- | --- |
| PC | 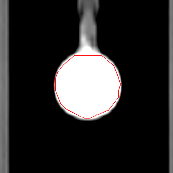 | 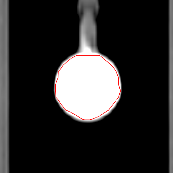 | 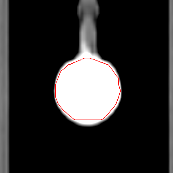 | 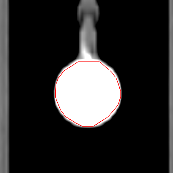 | 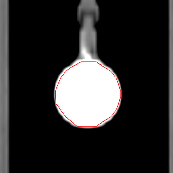 | 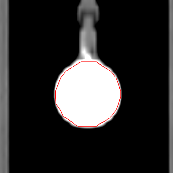 | 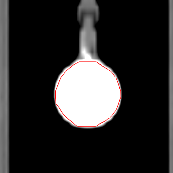 | 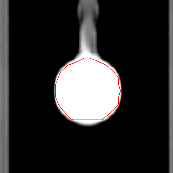 | 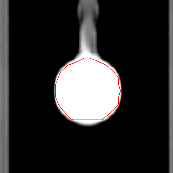 | 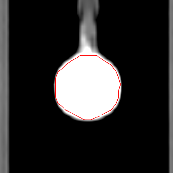 |
| SC | 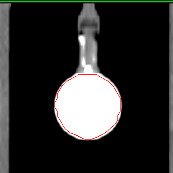 | 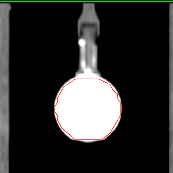 | 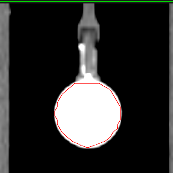 | 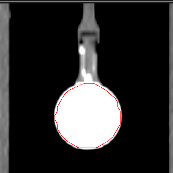 | 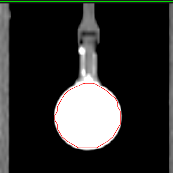 | 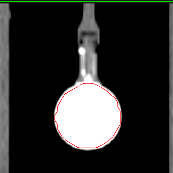 | 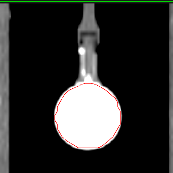 | 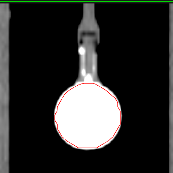 | 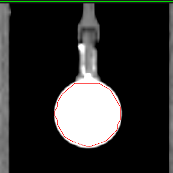 | 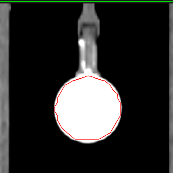 |
| SI | 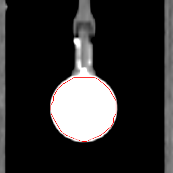 | 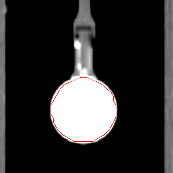 | 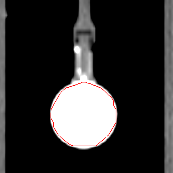 | 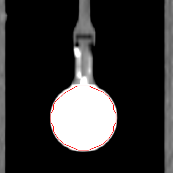 | 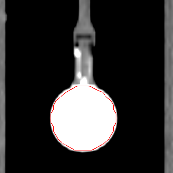 | 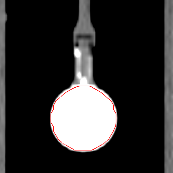 | 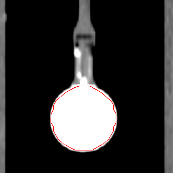 | 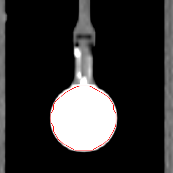 | 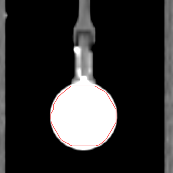 | 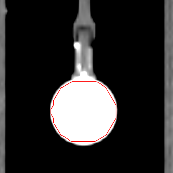 |

Table S4: individual phases for double amplitude (DA) breathing curve acquired on Pconv, Sconv and Si4D.

| DA | 0% | 10% | 20% | 30% | 40% | 50% | 60% | 70% | 80% | 90% |
| --- | --- | --- | --- | --- | --- | --- | --- | --- | --- | --- |
| PC | 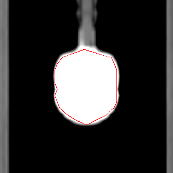 | 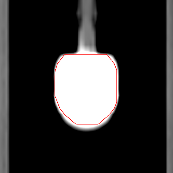 | 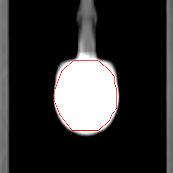 | 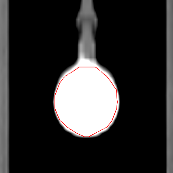 | 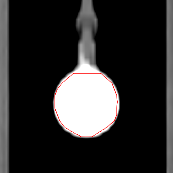 | 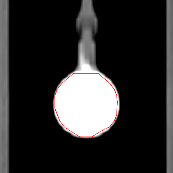 | 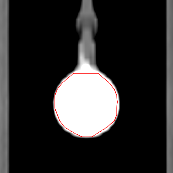 | 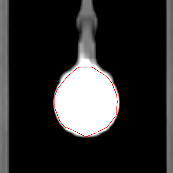 | 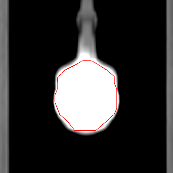 | 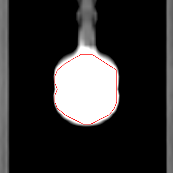 |
| SC | 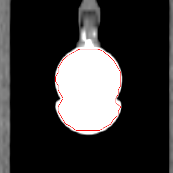 | 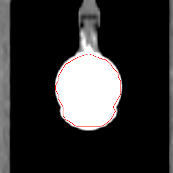 | 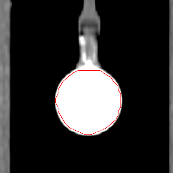 | 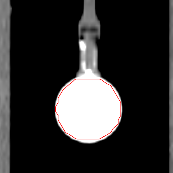 | 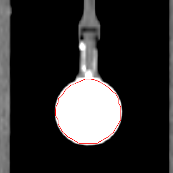 | 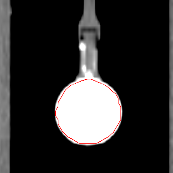 | 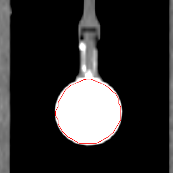 | 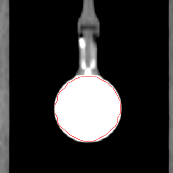 | 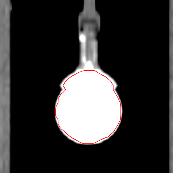 | 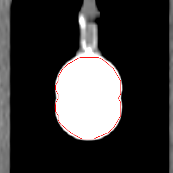 |
| SI | 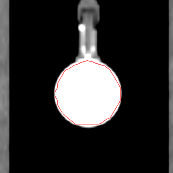 | 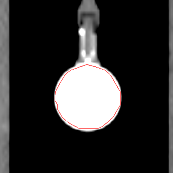 | 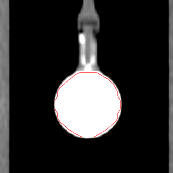 | 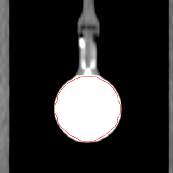 | 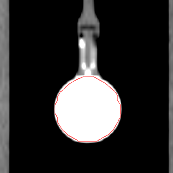 | 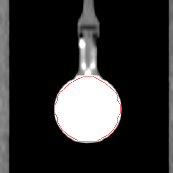 | 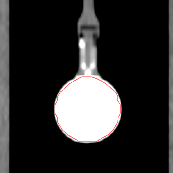 | 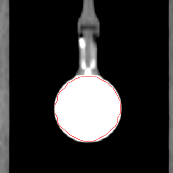 | 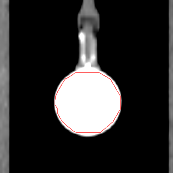 | 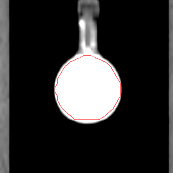 |

Table S5: individual phases for irregular frequency (IF) breathing curve acquired on Pconv, Sconv and Si4D.

| IF | 0% | 10% | 20% | 30% | 40% | 50% | 60% | 70% | 80% | 90% |
| --- | --- | --- | --- | --- | --- | --- | --- | --- | --- | --- |
| PC | 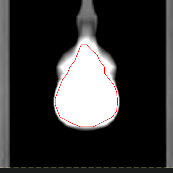 | 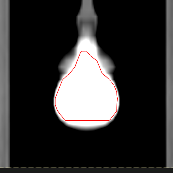 | 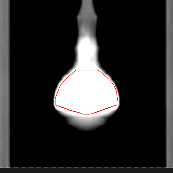 | 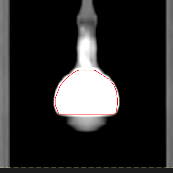 | 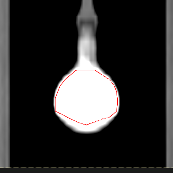 | 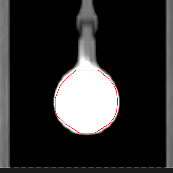 | 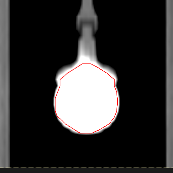 | 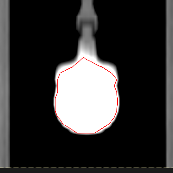 | 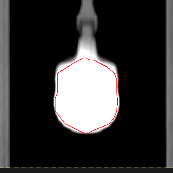 | 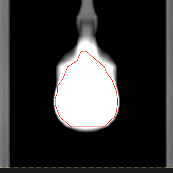 |
| SC | 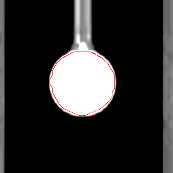 | 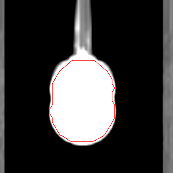 | 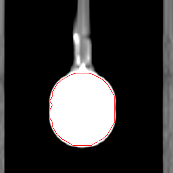 | 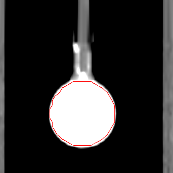 | 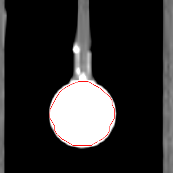 | 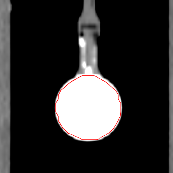 | 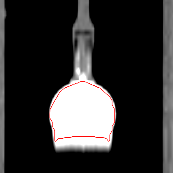 | 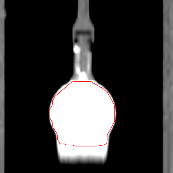 | 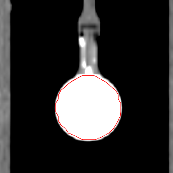 | 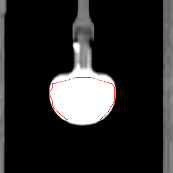 |
| SI | 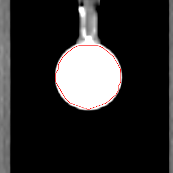 | 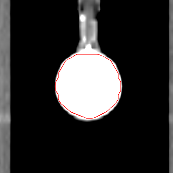 | 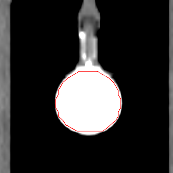 | 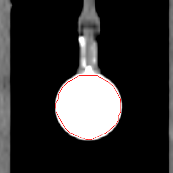 | 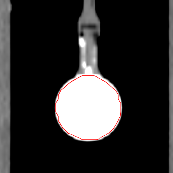 | 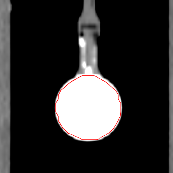 | 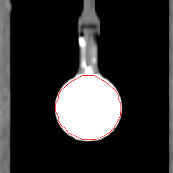 | 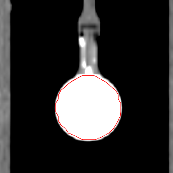 | 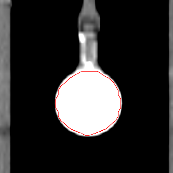 | 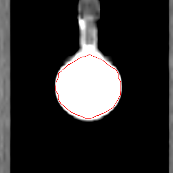 |
